# In situ optical measurement of particles in sediment plumes generated by a pre-prototype polymetallic nodule collector

**DOI:** 10.1038/s41598-024-72991-y

**Published:** 2024-10-12

**Authors:** Souha El Mousadik, Raphael Ouillon, Carlos Muñoz-Royo, Wayne Slade, Chuck Pottsmith, Thomas Leeuw, Matthew H. Alford, Ole A. Mikkelsen, Thomas Peacock

**Affiliations:** 1 Massachusetts Institute of Technology, 77 Massachusetts Avenue, Cambridge, 02139 USA; 2 Sequoia Scientific, Inc., 2700 Richards Road, Bellevue, 98005 USA; 3 Scripps Institution of Oceanography, University of California San Diego, 8622 Kennel Way, La Jolla, 92037 USA

**Keywords:** Sediment plume, In situ particle size distribution, Deep-seabed mining, Physical oceanography, Ocean sciences

## Abstract

This study presents in situ, high-resolution optical measurements of particle size distributions (PSD) within sediment plumes generated by a pre-prototype deep seabed nodule collector vehicle operating in the abyssal Pacific Ocean. These measurements were obtained using a cutting-edge instrument, the LISST-RTSSV sensor. The data collected in situ reveal marked differences compared to previously reported laboratory-based, ex situ measurements. The grain size and other key particle shape characteristics are found to be dependent on multiple factors, including the collector vehicle maneuvers, the time elapsed following sediment discharge, and the complex hydrodynamic processes that generate the sediment in suspension. Significantly, the PSD from a highly complex succession of straight-line maneuvers converges to that of the canonical case of a simple straight-line driving maneuver within a timescale of ten minutes. Our results underscore the importance of parameterizing sediment plume transport models based on well-informed, comprehensive PSDs of detrained suspended sediment measured in situ at adequate timescales and in regions no longer strongly influenced by active and complex hydrodynamic processes.

## Introduction

Interest in deep-seabed mining is increasing, driven by the surging demand for critical minerals such as cobalt and nickel. It is of utmost importance to fully understand, a priori, the potential environmental impacts and effects associated with proposed seabed mining activities and address research gaps^1,2^. In the case of polymetallic nodules, which is the focus of this paper, the process of separating and collecting the deposits from the topmost sediment layer of the seabed in the form of accretions will result in the suspension of sediment discharged in the wake of the nodule collecting vehicle, creating an extended disturbance across the seabed^3–5^. Furthermore, nodule mining may create a secondary plume resulting from the discharge of sediment-laden water at some depth in the water column or just above the seabed after the nodules have been transported to the surface platform^6,7^. Similar plume concerns have been raised for other forms of proposed seabed mining, such as Seafloor Massive Sulfides and Cobalt Crusts^8^.

Recent results on nodule collector plume dynamics for systems using a standard hydrodynamic pickup mechanism show that the spreading of the collector-generated sediment plume is initially driven by negative buoyancy of the sediment-laden mixture following the discharge. This results in a low-lying turbidity current spreading laterally from the collector vehicle^5^. During this process, some of the discharged sediment is deposited locally, whilst the remaining sediment is detrained from the turbidity current and is then available for far-field transport. The initial composition and concentration of the detrained sediment remaining in suspension are set by a complex interaction of turbulent mixing processes inherent to the turbidity current, the turbulent structure of the wake behind the collector vehicle, the background ocean currents, and the ambient ocean turbulence. The subsequent passive transportation of this suspended sediment away from the collector vehicle mining site is governed by the combination of advection in the direction of the background currents, vertical settling of sediment, and turbulent diffusion in the plane normal to the advection direction^5,9^. The initial height of the detrained sediment and the sediment settling properties are key factors in setting both the maximum vertical extension and the overall residence time of the plume^4^. Specifically, contemporary model predictions of the extent of such plumes vary by orders of magnitude (e.g., 1–100 km) for different inputs of sediment particle size distributions (PSD) and associated settling velocity distributions (SVD)^4^.

Characterizing the sediment PSD and morphology is a critical component in constructing an SVD, with the physical structure of the initial sediment that is detrained being set by disturbance-specific and highly complex fluid and particle dynamics. To date, however, characterizing marine sediment particles suspended by proposed deep seabed mining technology has been hindered by inherent challenges associated with established methods for sampling and quantifying such particle aggregates^10–12^. Most of the current knowledge pertaining to deep-seabed sediment particles is based on inherently disruptive sampling and ex situ sizing methods. Ex situ particle sizing methods, for example, introduce significant uncertainties through the loss and disruption of particles by the collection methods employed, with any handling likely to cause breakage or aggregation of the particles. Common procedures to obtain water samples through Niskin bottle sampling have been reported to lead to a reduction in the maximum particle size by an order of magnitude due to the breakage of large aggregates, with the problem being further exacerbated by incomplete extraction of fast-settling particles^13–15^. Defects and loss of seal in Niskin bottle closures are known to lead to water leaking during the ascent, leading to a further loss of larger particles that have settled during the retrieval process^12^. Samples obtained by pumping techniques, including peristaltic pumps, are considered to produce sufficient shear to break flocs^16^. Further uncertainties for ex situ methods stem from sample transport and storage, during which both aggregation and disaggregation can occur, whereby the longer a sample is allowed to sit before retrieval, the less the sample quality and reliability can be guaranteed^14^. Additional particle sizing uncertainties can be specific to the ex situ size analysis technique employed. For example, this is the case for particle size distributions measured from sediment samples prepared from an oven-dried aliquot, collected by means of multi-corers and then re-suspended through different stirring techniques^17^; in this case, particle sizing uncertainties stem from the sample preparation protocol itself.

Another key consideration is the necessity to obtain data for sediment disturbances created by realistic mining technology, as the nature of the suspended sediment is likely to be technology-dependent. While several studies have sought to assess, parameterize and model the impacts of deep-seabed mining^3,9,18–24^, except the Deep Ocean Mining Environmental Study (DOMES) project^9,18^, none of these studies monitored sediment plumes generated by deep seabed mining technology. Furthermore, not only were in situ measurements of the suspended sediment properties not possible, but in many cases, there were difficulties in obtaining sufficiently concentrated samples and poor representation of the samples post-collection due to sample handling, storage, and analysis^9^. For the DOMES project, the sediment settling velocity distribution was inferred a posteriori by fitting the obtained field data to a simple, time-dependent plume model that assumed suspended particulate matter followed passive advection, settling, and diffusion, and treated the settling velocity distribution and vertical diffusivity as parameters^9^. However, the accuracy of results obtained for the settling distribution in such a manner remains highly uncertain, given that initial plume conditions at the time of this study were not known.

We sought to address the aforementioned shortcomings and uncertainties through the use of a noninvasive and nondestructive in situ particle sizing instrument deployed during a realistic collector mining vehicle trial. We developed and utilized the LISST-RTSSV (Real Time Size and Settling Velocity) instrument that can operate at abyssal depths down to 6000 m. Details pertaining to the LISST-RTSSV instrument design are described further in the “Materials and methods” section. In Spring 2021, the Belgian contractor Global Sea Mineral Resources NV (GSR) deployed a deep-seabed nodule mining pre-prototype collector vehicle (henceforth referred to as collector vehicle) in the Clarion Clipperton Zone (CCZ) with the LISST-RTSSV mounted. For reference, GSR’s pre-prototype collector, named Patania II, employs the same collection technology as the proposed full-scale commercial system but is of limited width. This was the first time such technology had been tested in the CCZ. These collector trials offered an unprecedented opportunity for the LISST-RTSSV to investigate in situ the nature of suspended sediment mobilized during a realistic collector vehicle operation.

**Fig. 1:**
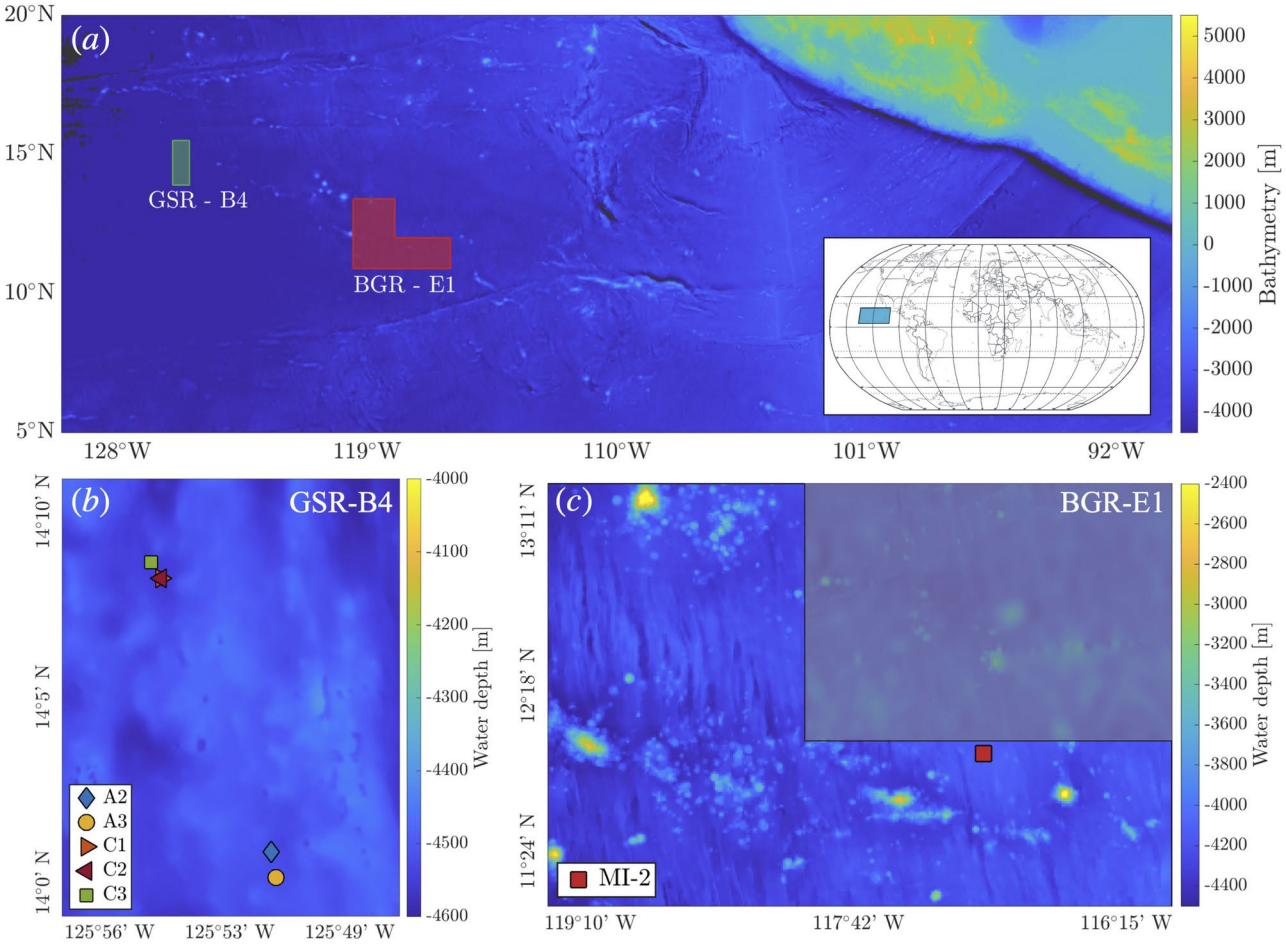
(**a**) Bathymetry map showing the two areas GSR-B4 and BGR-E1, in green and red respectively, located in the CCZ where the collector trials were conducted. GSR-B4 is contracted by Global Sea Mineral Resources NV (GSR, Belgium), and BGR-E1 by the Federal Institute for Geosciences and Natural Resources (BGR, Germany). (**b**) Bathymetry of the GSR-B4 area of operation and the locations of the *selfie* experiments. (**c**) Bathymetry of the BGR-E1 operation area and the mining maneuver experiment site, where the European Mining Impact 2 research project was conducted (MI-2). The GSR-B4 bathymetry is provided by Global Sea Mineral Resources NV, and the BGR-E1 bathymetry is acquired from the GEBCO 2022 Grid, GEBCO Compilation Group (2022).

For the experiments described in this paper, two qualitatively different collector maneuvers were carried out on the seabed in two regions of the CCZ (Fig. 1-a). The first type of maneuver, termed a *selfie*, was performed in the GSR-B4 polymetallic exploration area (Fig. 1-b) for the purpose of directly measuring the turbidity current phase of the sediment plume generated during nodule collection. Extensive details about this study and in-depth insight into the turbidity current behavior of the collector-generated sediment plumes are presented in^5^. The second type of maneuver, referred to as a *mining pattern*, was performed in the polymetallic exploration area BGR-E1 contracted by the German Federal Institute for Geosciences and Natural Resources (BGR), and carried out an impact experiment in parallel with the European Mining Impact 2 research project (MI-2) (Fig. 1-c). This maneuver comprised driving multiple parallel lines, during which the collector picked up the nodules to simulate a sea-bed mining operation scenario, albeit at a smaller scale. Throughout all these maneuvers, the LISST-RTSSV was mounted at the rear of the nodule collector vehicle at a height of $\sim$5m above the seabed (Fig. 8). In this paper, we begin by presenting the particle size distribution results derived from the *selfie* experiments, elucidating observations within the framework of the governing fluid dynamic processes responsible for sediment suspension. Subsequently, we delve into measurements acquired during the *mining pattern* across different collector vehicle operations. Finally, we compare the *mining pattern* results against those made in the *selfie* experiments, culminating in a discussion of our findings and their implications for monitoring deep seabed mining sediment plumes. An objective of this work is to build upon and advance recent work^5^ by investigating and characterizing the size and morphology of the sediment suspended in the wake of turbidity currents created by simple and complex nodule collector operations.

## Results

### *Selfie* maneuvers

**Fig. 2:**
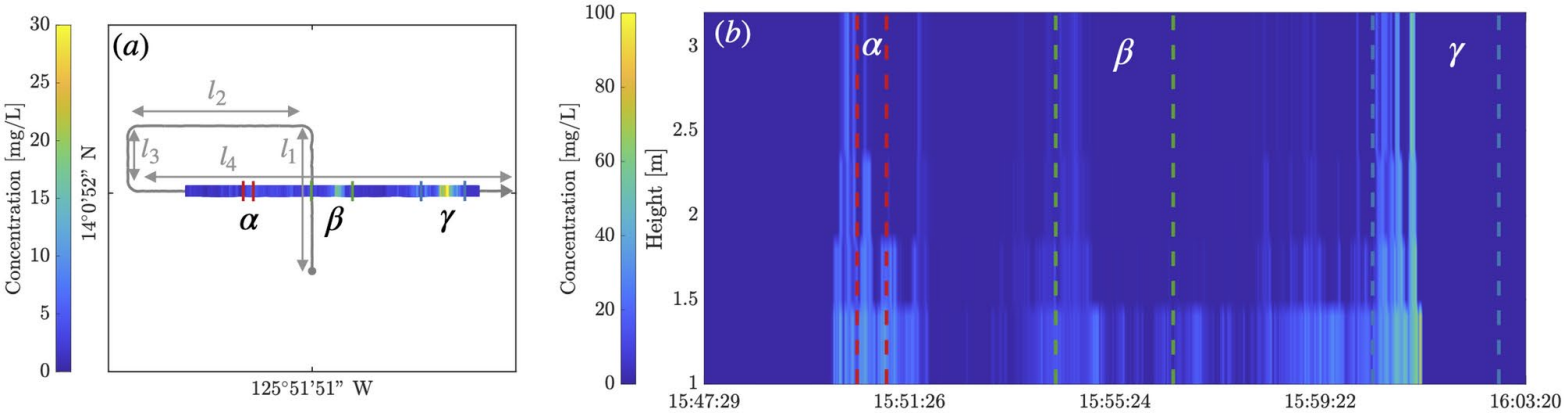
(**a**) Top view of the trajectory of *selfie A*2, with LISST-RTSSV sampling locations $\alpha$, $\beta$, and $\gamma$ indicated: $\alpha$ is the intersection of the collector vehicle with the first sediment plume front, $\beta$ is the crossing intersection with the collector vehicle tracks, and $\gamma$ is the intersection of the collector vehicle with the second plume front. The color map in (**a**) corresponds to the averaged sediment concentration (in mg/L) measured with the four calibrated Seapoint Turbidity Meter (type S) sensors positioned in the vicinity of the LISST-RTSSV at the back of the collector vehicle at 5 m above the seabed. (**b**) Shows a color plot of the vertical sediment concentration, as a function of time, measured while driving the final *selfie* segment $l_4$. The vertical dashed lines indicate where data was collected by the LISST-RTSSV. The sediment concentration in (**b**) is obtained from calibrated STM-S sensors mounted on a pole at the front of the collector vehicle at altitudes of 1 m, 1.6 m, 2 m, 2.5 m, and 3.2 m, as shown in Fig. 8-a. The concentration measurements are linearly interpolated in the vertical direction. The STM-S calibration curves for suspended sediment are given in Fig. S2.

The *selfie* maneuvers, an example of which is shown in Fig. 2-a, comprised four main segments. During the first segment, $l_1\sim$100 m, the collector vehicle was actively collecting polymetallic nodules, resulting in sediment being discharged in the vehicle’s wake. Upon completion of this segment, the collection system was turned off, thereby ceasing the discharge. Then, the collector proceeded to conduct three turns with intervening driving segments $l_2$ and $l_3$, to position itself at the start of segment $l_4$. Moving along segment $l_4$, the collector drove perpendicularly to segment $l_1$, so that it sampled a cross-section of the plume it generated during the nodule collection. A total of eight *selfie* experiments were conducted across three different sites within the GSR-B4 exploration area. The length of segment $l_2$ and the collector vehicle speed were varied across selfies to intersect the plume at different times in its evolution, thereby obtaining a comprehensive picture of plume evolution across multiple experiments. A complete investigation into the behavior of the collector-generated sediment plumes pertaining to this study can be found in Ref.^5^.

The LISST-RTSSV collected data during five of the *selfie* experiments conducted at two sites (locations marked *A* and *C* in Fig. 1-b), operating in a pre-scheduled burst/sleep mode, which is necessary to meet power and memory usage requirements based on the duration of the pre-planned collector vehicle operations at the seabed. In this mode, the instrument was sampling continuously during the burst phase for three minutes, followed by a three-minute sleep phase. Selfies *A*2 and *A*3 collected images at a sampling rate of 4 Hz, which was later increased to 10 Hz for *C*1, *C*2, and *C*3 to capture blurry particles better. Further details on the sampling procedure are in the “Materials and methods” section.

In Fig. 2, corresponding to *selfie* *A*2, we identify three key points of interest during a *selfie* maneuver: point $\alpha$ corresponds to where the collector vehicle first encountered the sediment plume front, point $\beta$ is where the collector vehicle crosses its previous tracks, and point $\gamma$ corresponds to where the collector vehicle exited the plume. In Fig. 2-b, two plume fronts, $\alpha$, and $\gamma$, are apparent where sediment concentration exceeded 50 mg/L and reached a few meters above the seabed, corresponding to the collector vehicle having entered and exited the body of the laterally spreading turbidity current. Between $\alpha$ and $\beta$, and between $\beta$ and $\gamma$, the sampled plume was observed to be low-lying, typically below the collector height, and the sediment concentration was a few mg/L. Positioned at a height of 5m, the sediment suspension sampled by the LISST-RTSSV for these three regions is expected to have been suspended material that was detrained from the body of the actively spreading turbidity current plume. However, this is not expected to be the case for position $\beta$, where the collector vehicle crossed its previous tracks. In this location, the sediment is understood to have been directly suspended by a different fluid dynamic process, that being the turbulent wake of the collector vehicle^5^.

**Fig. 3:**
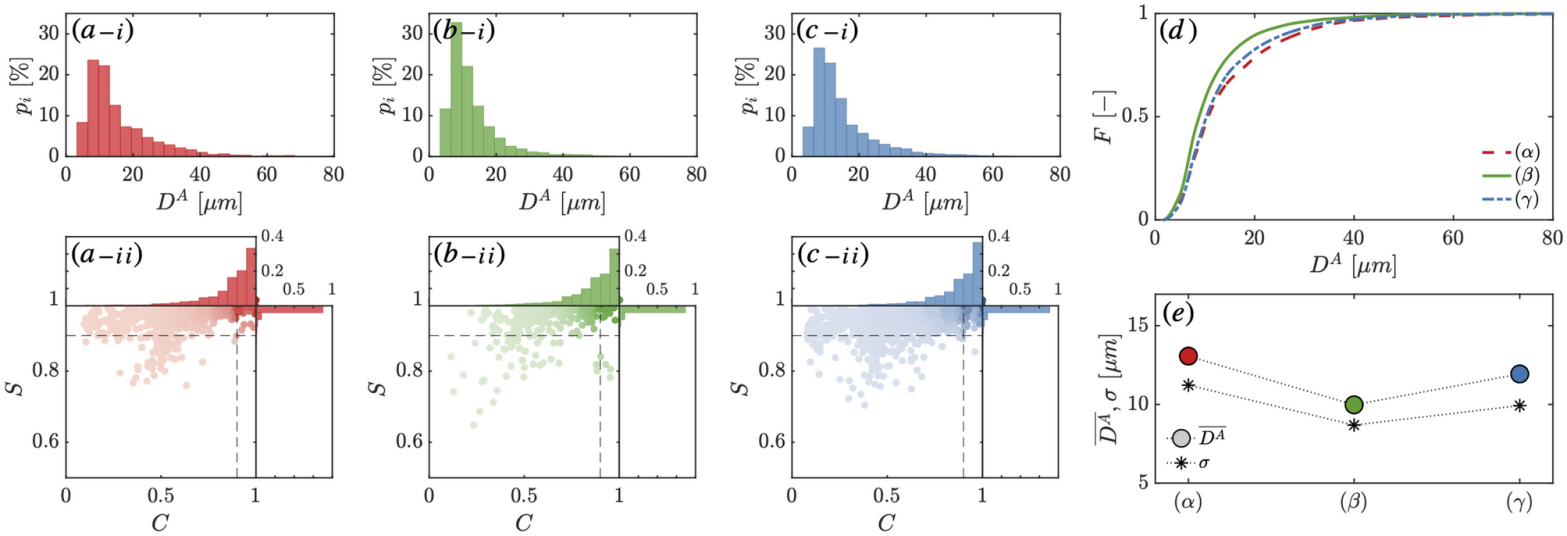
(**a**–**i**, **b**–**i** and **c**–**i**) show the number-based particle size distributions $p_i$ for *selfie A*2, shown in Fig. 2, corresponding to the sampling locations $\alpha$, $\beta$, and $\gamma$, respectively. (**a**–**ii**, **b**–**ii** and **c**–**ii**) present the measured shape descriptors, solidity *S* versus circularity *C*, for points $\alpha$, $\beta$, and $\gamma$, with associated histograms. (**d**) The cumulative number-based size distribution *F*, for each of the sampling locations, represents the probabilities of particles of size under $D^A_i$. (**e**) The mean particle diameter $\overline{D}^A$ and the distribution standard deviation $\sigma$ for each of the sampling locations are shown.

To clarify the discussions around particle size in this paper, the particle size distributions (PSDs) presented henceforth have been explicitly defined in the “Materials and methods” section. Figure 3-(a-i)–(c-i) present the number-based particle size distribution $p_i$ for points $\alpha$, $\beta$, and $\gamma$ in *selfie* *A*2, respectively, as indicated in Fig. 2. All three measured PSDs exhibit a clear large-particle tail with positive skewness and narrow, leptokurtic distribution, centered around a mode equal to 4.9 $\upmu$m.

We observe marked differences in the number-based cumulative size distribution, *F*, and the mean particle diameter $\overline{D}^A$, between plume entry and exit points $\alpha$ and $\gamma$ and the track crossing point $\beta$ (Fig. 3-d,e, respectively). Point $\beta$ showed a $D_{50}$ = 8.8 $\upmu$m whereas points $\alpha$ and $\gamma$ had a $D_{50}$ = 10.7 $\upmu$m and $D_{50}$ = 10.4 $\upmu$m respectively. Point $\beta$ was also characterized by a narrower distribution with a higher kurtosis $\mu _4$ = 19.4 and positive skewness $\mu _3$ = 3.15 compared to points $\alpha$ and $\gamma$ which registered a kurtosis value equal to $\mu _4$ = 11.1 and $\mu _4$ = 10.2, respectively, and a positive skewness equal to $\mu _3$ = 2.27, respectively. These differences can be attributed to the distinct fluid dynamic processes governing the sediment suspension at these locations. The sediment measured at points $\alpha$ and $\gamma$ was detrained from the turbidity current during its propagation and remained suspended in its trailing wake at a height of at least 5 m. However, the suspended sediment at point $\beta$, which was at distinctively lower abundance and sediment concentrations than points $\alpha$ and $\gamma$, resulted from vertical mixing due to wake turbulence generated by the collector and did not become part of the turbidity current. Of note, in Fig. 2, we find that the turbidity meters positioned at the rear of the vehicle at 5 m detect higher sediment concentrations than the highest positioned turbidity meter at 3.2 m at the front of the collector, but lower concentrations than the turbidity meter positioned at 2.5 m. This implies that the sediment sampled by the LISST-RTSSV at 5 m is likely sediment originating from heights between 2.5 and 3 m, perturbed upward by the collector as it travels through its own disturbance. Nevertheless, we are able to discern quantitative differences in the sediment detrained from the main body of the current and that in suspension in the wake of the collector tracks. At such low sediment concentrations $\sim \mathcal {O}$ (30 mg/L), the perturbation caused by the collector vehicle is unlikely to lead to flocculation, thereby preserving the original sediment composition^17,25^.

A benefit of optical imaging is that shape features can also be analyzed to complement particle size information. We choose circularity and solidity to describe the particle shape in the two-dimensional plane, assumed to reflect the three-dimensional shape fairly reasonably for a sufficient number of frames and particles. Circularity, defined as $C = 4 A/\pi D^2_F$, is a macroscale parameter indicating how closely the shape of an object approaches that of a circle by comparing the area *A* of the detected particle to that of a disk of diameter $D_F$, the maximum Féret diameter^26,27^, defined to be equal to unity for a perfect circle. Solidity *S* is a mesoscale indicator of the particle contour’s rugosity within the imaging system’s resolution, which is 1 $\upmu$m for the LISST-RTSSV instrument. It is defined as the ratio of the area of an object to the area of its convex hull, taking a maximum value of 1 for a solid convex object and less than 1 for objects with irregular boundaries or holes^26^. The measured shape descriptors, solidity *S* versus circularity *C*, for locations $\alpha$, $\beta$, and $\gamma$ are presented in Fig. 3-(a-ii)–(c-ii), respectively.

Particle shape information becomes critical for two reasons when PSD measurements are used to infer particle settling velocity based on existing settling velocity models incorporating particle shape and density information. First, the shape can significantly influence the settling dynamics of particles, with a departure from spherical shapes typically leading to slower particle settling velocity^28–30^. Second, the particle size herein has been defined based on the equivalent projected area diameter $D^A$, where the projection of the particle perpendicular to the plane of view is assumed to be circular. This assumption will result in overestimating the particle size in cases of very elongated or flaky particles due to such particles being preferentially oriented such that the largest dimension is within the imaging field view. Generally, such cases have been attributed to the presence of flocs^31^, which are characterized by shape descriptors that deviate significantly from the canonical solid sphere assumption. For these cases, the need for measurements of shape features, e.g., circularity and solidity, becomes not only complementary but imperative.

Here, the data in the lower left quadrant in Fig. 3-(a-ii)–(c-ii), where *C* and *S* are smaller than 0.9, indicate that irregular particles are indeed present in the population of the sampled particles. However, the bulk of the data is indeed situated in the upper right quadrant, where we find that more than $90\%$ of the sampled particles have a solidity value higher than 0.9, indicating that, within the resolution of the system, the majority of the particulates had regular boundaries, with less than $0.1\%$ exhibiting significant angularity. And more than $53\%$ of the sampled particles have a circularity between 0.9 and 1, with more than $89\%$ of the sampled particles having a circularity larger than 0.7, indicating that it is acceptable to the first order at least, to use the equivalent projected area diameter $D^A$ as a reasonable size descriptor in the discussions that follow.

We find that solidity and, to some extent, circularity decreased from point $\alpha$ to point $\gamma$, highlighting the increased presence of irregular and non-circular particulates in the sampled water volume as time evolved. This observation is consistent with a correlation between the particle shape and the settling rate, as more circular and solid particles are expected to settle faster than their irregular counterparts and, therefore, be increasingly absent from the regions being sampled as time passes. Particles sampled at location $\beta$ were notably more circular and solid, thereby less irregular, than those sampled at locations $\alpha$ and $\gamma$. This finding correlates with the presence of finer sediment particles at $\beta$ and further supports the distinction between sediment detrained from the gravity current and sediment suspended by vertical mixing directly in the intense wake of the collector.

**Fig. 4:**
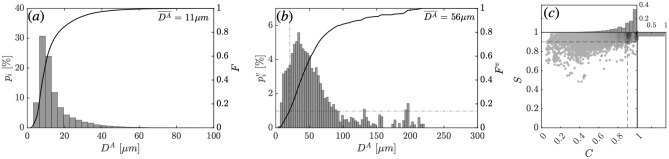
(**a**) and (**b**) show the number-based particle size distribution $p_i$ and the volume-based particle size distribution $p^v_i$ corresponding to all five conducted selfies combined. The dashed lines in (**b**) show that 88% of the sediment mass is accounted for by particles of size greater than 21 $\upmu$m. (**c**) Shows the measured shape descriptors, solidity *S* versus circularity *C*, with associated histograms, for all the five realized selfies combined.

Data from all the selfies conducted were analyzed in a similar manner to that presented in detail for *selfie* *A*2. The results pertaining to each of the five *selfie* experiments are given in Figs. S3 and S4 in the Supplementary Information. Due to the necessary burst sampling mode applied during data collection and the presence of some blurry sediment particles due to fluid motion in the imaging chamber, we could not obtain data at all key points $\alpha$, $\beta$, and $\gamma$ for all the selfies. Figure 4-a presents the number-based probability distribution $p_i$ across all locations for the five realized selfies, such that all particles from every frame of every *selfie* have been considered, for a total number of $N_p = 7.6 \times 10^4$ particles. The resulting number-based particle size distribution exhibits a positive skewness ($\mu _3$ = 3.28), a very high kurtosis ($\mu _4$ = 24.5), and a distribution standard deviation $\sigma$ = 10.3 $\upmu$m, with fine particulates thus representing the overwhelming majority of particles by count. In addition, significant narrowness of the distribution is found, with a dominant mode located at 4.9 $\upmu$m. The median particle size is $D_{50}$ = 9.5 $\upmu$m, and the diameter at the $90\%$ point in the cumulative (undersize) particle size distribution is equal to $D_{90}$ = 25 $\upmu$m, indicating the presence of an abundance of primarily fine and medium silt particles.

Although small particles were present in great abundance compared to larger particles, it is important to note that this does not necessarily translate to volume-based quantities. Given that many quantities of interest, such as sediment concentration and sedimentation mass flux, depend on the volume (or mass) of the suspended sediment, it is also important to consider the volume-based particle distribution. Figure 4-b presents the volume-based probability distribution, $p^v_i$, resulting from the conversion of the number-based distribution $p_i$ in Fig. 4-a. The volumetric probability distribution conversion method is detailed in the “Materials and methods” section. Here, the height of each bar represents the percentage of the total particle volume occupied by particles whose size lies within the range of that bin. The volume-based distribution exhibits a kurtosis equal to $\mu _4 = 5.6$, with a standard deviation $\sigma$ = 45.3 $\upmu$m characterizing the broad spread of the data. The distribution remains positively skewed with a mode centered around 30.8 $\upmu$m, with a significantly lower skewness value $\mu _3 = 1.7$, and a significantly increased median particle size $D_{50}$ = 44.5 $\upmu$m. Effectively, $p^v_i$ highlights better the presence and contribution of the coarse particles, which are likely to settle fast, as we find that more than $88\%$ of the suspended sediment mass is occupied by particles larger than 21 $\upmu$m. Thus, we find that only $18\%$ of the total number of particles contributed significantly to the total suspended sediment mass.

Regarding particle shape descriptors, shown in Fig. 4-c, we find that less than $0.1\%$ of the population exhibits significant angularity, highlighting once more that the majority of the particulates had regular boundaries. Moreover, more than $52\%$ of the sampled particles have a circularity between 0.9 and 1, with around $86\%$ of the sampled particles having a circularity larger than 0.7, similar to the singular case of *selfie* *A*2.

### *Mining pattern* maneuver

**Fig. 5:**
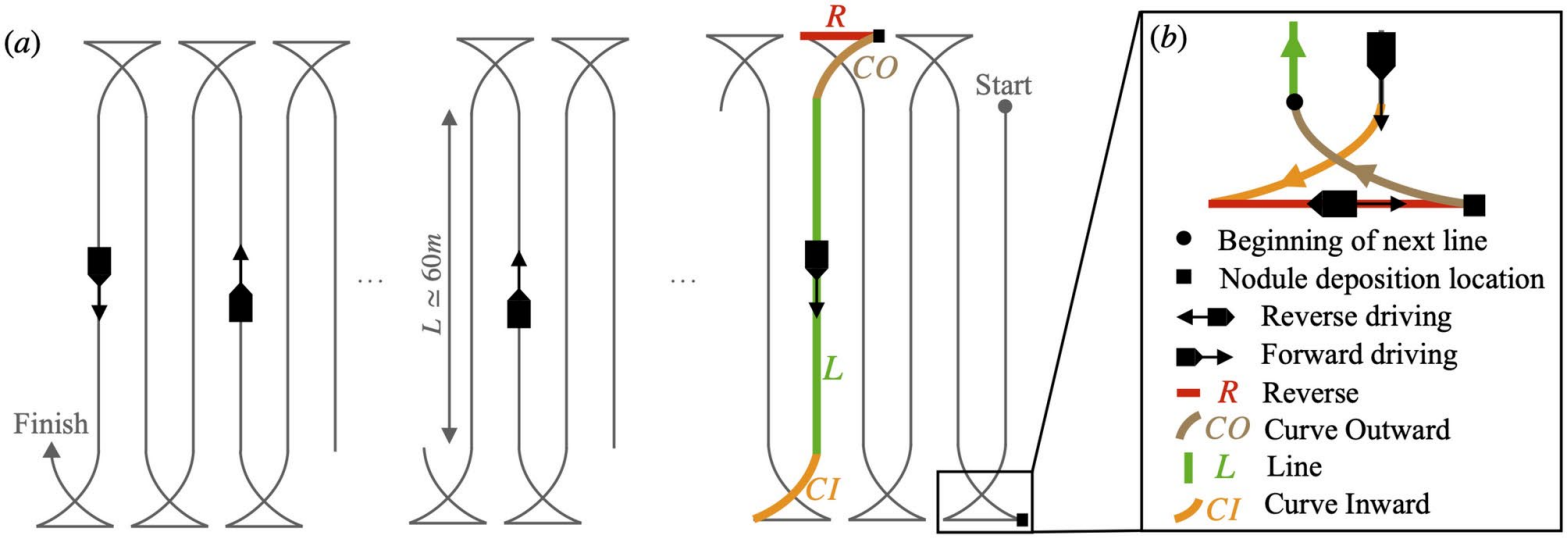
(**a**) Schematic illustrating the *mining pattern* maneuver. The collector drove consecutive parallel straight lines $L\simeq$ 60m, during which the collector’s pumps were operated and the polymetallic nodules collected. At the end of *L* (in green), the collector was re-positioned by driving in a curve, *CI* (in orange), to begin the turning maneuver. (**b**) Illustration of the turning maneuver. After the entry maneuver *CI*, the nodules were deposited at the end of the reverse driving segment *R* (in red), followed by the collector outwardly driving in a curve, *CO* (in brown), to the location where the next line segment *L* begins.

**Fig. 6:**
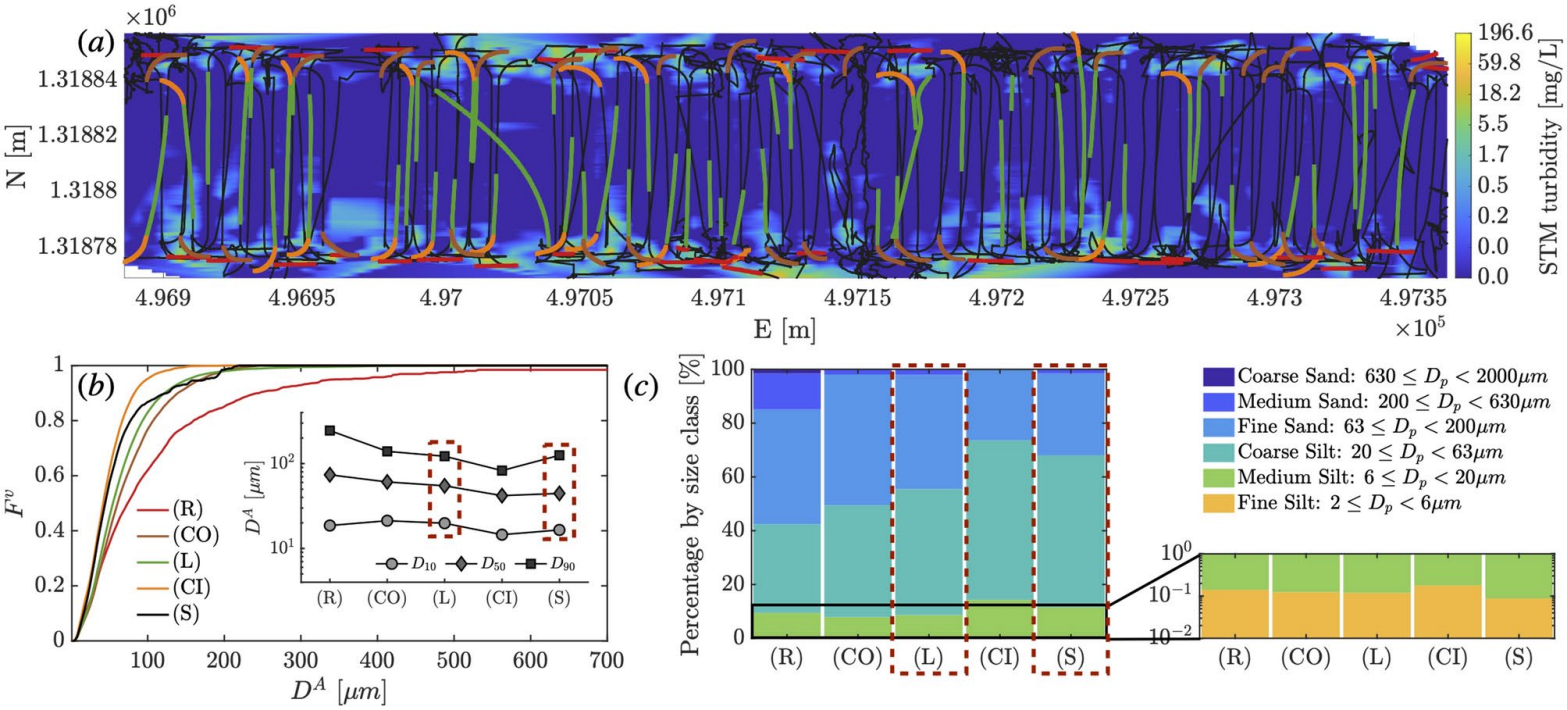
(**a**) Top view of the collector vehicle trajectory. The color map corresponds to the sediment concentration measured by the lowest mounted Seapoint Turbidity Meter (STM-S) at the front of the collector, linearly interpolated on a grid of $\Delta x$ = 5 m and $\Delta y$ = 0.1 m. Colored data points corresponding to the four data categories during the vehicle operations (*R*, *CO*, *L*, and *CI*) are shown. (**b**) Shows the cumulative volume-based particle size distribution $F^v$ of the sampled suspended sediment generated by the different operations during the *mining pattern* maneuvers and during the selfies. In the insert, $D_{10}$, $D_{50}$ and $D_{90}$ are plotted for each of the four mining impact data categories and the combined selfies data, corresponding to the $10\%$, $50\%$ and $90\%$ points in the cumulative undersize particle size distribution. (**c**) Presents the average percentage of sediment within each particle size class during the *mining pattern* maneuvers and the selfies. A magnified view of the finest grain classes is shown on the right. The grain size classes are based on the ISO 14688-1 classification scale.

The *mining pattern* maneuver was conducted in the contract area BGR-E1 shown in Fig. 1-c. This maneuver, illustrated in Fig. 5, comprised driving multiple 60m-long parallel lines, during which the collector picked up nodules. The nodule load was then deposited at the end of each line before starting the next one, following the schematic in Fig. 5-b. During these maneuvers, the LISST-RTSSV operated in the burst/sleep mode once again to meet power and memory requirements based on the scheduled vehicle operations. Images were collected at a sample rate of 2 Hz to meet memory requirements. The instrument collected data for over 30 h, repeatedly alternating between a burst phase of 5 min and a 10-min sleep phase. Correlating between the collector vehicle trajectory and the availability of data from the LISST-RTSSV resulted in the classification of the collected data into four distinct data categories based on suspended sediment origin: data collected during the reverse driving maneuver *R*, data collected during the outward driving curve *CO* following the reverse driving, data collected driving along the straight line segment *L*, and finally data collected through the inward driving curve *CI* prior to positioning for the next reverse maneuver. Figure 5 highlights each of the different data categories, and Fig. 6-a shows all of the four different identified sampling scenarios superimposed onto a plot of the actual collector vehicle trajectory. The background color map in Fig. 6 corresponds to the suspended sediment concentration measured by the lowest positioned turbidity meter, located at 1 m above the seabed at the front of the collector (Fig. 8-a).

The cumulative volume-based particle size distributions $F^v$ of the sampled suspended sediment generated by the different operations are presented in Fig. 6-(b), and we provide the comprehensive results for each sampling scenario in Fig. S5. In Fig. 6-(c), we present the average percentage of sediment by volume within each particle size class, based on the ISO 14688-1 classification scale, for each of the *mining pattern* maneuvers. Although particle grains smaller than 2 $\upmu$m are not accounted for here due to the imaging system resolution, this limitation does not affect the analysis herein, as the following discussion is made in the context of suspension of matter by volume (and therefore by mass). The contribution towards the total volume of particulate matter in suspension by extremely fine sediment grains is negligible compared to the larger sediment grain sizes.

During the reverse maneuver *R*, the LISST-RTSSV, which was positioned at the back of the vehicle, mainly sampled sediment directly kicked up by the collector as the nodules were deposited at the end of the reverse maneuver. The average suspended sediment concentration recorded by the rear-mounted turbidity meter sensors during this maneuver was $\overline{C}$ = 23 mg/L, with a high standard deviation of $\sigma _{C}$ = 24 mg/L and maximum values reaching upwards of 196 mg/L. In this maneuver, the total mass of suspended sediment is mostly occupied by fine sand (63 $\upmu$m $\le$ D < 200 $\upmu$m) and coarse silt grains (20 $\upmu$m $\le$ D < 63 $\upmu$m) at 42.6$\%$ and 33$\%$ respectively. Medium sand grains (200 $\upmu$m $\le$ D < 630 $\upmu$m) accounted for 13.4$\%$ of the suspension. Some coarse sand grains, larger than 630 $\upmu$m, were captured during this maneuver and accounted for 1.6$\%$ of the total suspended particulate matter (by volume). During the reverse driving and the deposition of the nodules, the collector generates elevated levels of coarse particles, which are sampled shortly after their suspension and before significant settling had occurred, justifying the presence of exceptionally large particles ($\ge$ 630 $\upmu$m) in the sampled volume at the LISST-RTSSV height. The remaining 9.4$\%$ of the suspension is composed of medium and fine silt, of sediment grains smaller than 20 $\upmu$m.

While the collector vehicle conducted the outward driving curve *CO*, the sediment sampled most likely originated from advected sediment that was suspended during *R* and/or during prior nearby operations. Since the collector was effectively driving forward and away from *R*, the fast-settling coarse sediment encountered previously would have had a chance to settle well below the sampling height of the LISST-RTSSV. We thereby find here that the coarse sand grains fraction is zero, and the medium sand grains fraction decreased substantially and accounted for only 2.0$\%$ of the total suspension in volume. Since the sediment grain fractions are calculated with respect to the total mass in suspension, we register an increase in both the fine sand and coarse silt grains, which are now at 48.6$\%$ and 41.6$\%$. This increase is primarily due to the settling of sediment larger than 200 $\upmu$m, as medium and fine silt sediment fractions remained at around 7.9$\%$. We also note that the rate of increase in the coarse silt grains is higher than that of the fine sand, as a portion of the particles larger than 63 $\upmu$m has likely settled as well. During this maneuver, the average suspended sediment concentration was $\overline{C}$ = 16 mg/L, with a standard deviation of $\sigma _{C}$ = 17 mg/L, and maximum values recorded were upwards of 95 mg/L.

During segment *L*, the sediment suspension sampled at 5 m comprised detrained sediment from multiple turbidity currents generated by the collector vehicle at different times, in addition to potentially a non-trivial fraction of sediment advected from the *R* maneuver, beyond *CO*, by the background currents. As the collector drove along a line *L*, moving much faster than the ocean bottom currents and thus further away from the location of maneuvers *R* and *CO*, it would have encountered increasingly older suspended sediment from prior legs of the *mining pattern*. Here, the medium sand grains fraction was measured at 2.0$\%$ of the total suspension, which originated mainly in the first few meters of the line segment, in the vicinity of *R* and *CO*. The fine sand grains fraction was equal to 42.5$\%$, the coarse silt grains fraction was 46.9$\%$, and the medium and fine silt sediment fractions accounted for 8.6$\%$ combined. The average suspended sediment concentration along the line maneuver was $\overline{C}$ = 4 mg/L, with a standard deviation equal to $\sigma _{C}$ = 5 mg/L. The first 10 m of the line indicated a mean sediment concentration equal to $\overline{C}$= 3.6 mg/L ($\sigma _{C}$ = 5.2 mg/L), with a maximum value of 30 mg/L. Whereas the last 10 m of the line had a lower mean sediment concentration equal to $\overline{C}$= 1.5mg/L ($\sigma _{C}$ = 1.0 mg/L), with a maximum concentration equal to 4.8 mg/L. Figure S6 provides the particle size data collected during segment *L* for each line section. This decrease in concentration along the line is due to the collector first encountering suspended sediment detrained from the most recently generated gravity current from the prior leg of the *mining pattern* and sampling older suspended sediment as it continues driving along *L*.

As the collector conducted the final maneuver, *CI*, it encountered sediment smaller than 200 $\upmu$m, that was previously suspended during *R* and CO and has not settled yet, and/or sediment advected from previous nearby operations. For this maneuver, the fine sand grains fraction decreased significantly down to 26.4$\%$, which was accompanied by an increase in the coarse silt fraction, which now constituted 59.4$\%$ of the total suspension, and a slight increase in both the medium and fine silt sediment fractions, which accounted for 14.2$\%$ combined. Along this maneuver, the average suspended sediment concentration was $\overline{C}$ = 1.9 mg/L, with a standard deviation equal to $\sigma _{C}$ = 1.6 mg/L.

These results highlight the non-trivial analysis of the settling of suspended sediment in the context of active collector vehicle operations, where comparisons and interpretations of changes between size fractions need to occur with respect to each other and in the context of ongoing and past operations. As such, the particle size distribution, and thus the settling velocity distribution, are not only specific to particle suspension hydrodynamics as shown by the *selfie* data, but are also dependent on the type of collector vehicle operations analyzed in the context of the sediment encounter time.

## Discussion

In a recent study^5^, turbidity currents were shown to govern deep-sea mining sediment plume generation. The present work builds on these findings and advances the state-of-knowledge by establishing the first in situ characterization of the sediment suspended in the wakes of turbidity currents generated by nodule collector vehicles, which in turn provides the foundation for reliable sediment plume modeling. Our study emphasizes that the particle size distribution (Fig. 6), and thus the settling velocity distribution, are disturbance-specific and dependent on many factors, such as the collector vehicle operations, the complex hydrodynamic processes, and the time elapsed following discharge. Despite the inherent challenges of coordinating a pre-programmed data acquisition plan with a constantly changing schedule of collector vehicle performance, throughout these complex abyssal experiments, we identified distinct regimes, differences, and similarities within and across the various conducted collector maneuvers. In the *selfie* maneuvers, for example, we noted differences between the particle size distribution measured for the detrained sediment from the gravity current body compared to that from the turbulent wake of the vehicle. In the *mining pattern* maneuvers, we observed a marked difference in the particle size distribution depending on whether the collector is conducting one type of operation (e.g., driving along a line) or another (e.g., reverse maneuver). The PSD is a complex function of the position within the plume and time elapsed since discharge and is governed not only by the initial properties of the suspended seabed sediment but, most importantly, by the series of fluid dynamics processes that affect it following the initial discharge. As such, when considering model simulations of seabed mining operations, our findings highlight the importance of correctly initializing such models with a physics-informed PSD that is commensurate with the model being utilized. To date, ocean models that have been used to simulate seabed mining scenarios^32,33^, have considered advection, diffusion, and settling processes but ignored key processes such as the turbulent wakes behind a collector vehicle and buoyancy-driven flows. Such models can only use PSDs obtained in regions where the sediment transport is no longer actively affected by such complex hydrodynamic processes^4^.

**Fig. 7:**
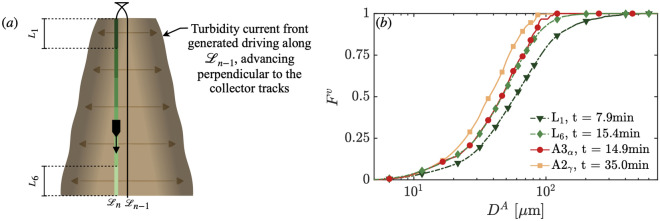
(**a**) Schematic showing the collector vehicle driving along line segment $\mathcal {L}_n$ intersecting the gravity current it previously generated along $\mathcal {L}_{n-1}$, the first and last 10m sections of the straight line $\mathcal {L}_n$ are denoted $L_1$ and $L_6$. When in $L_1$, the collector intersects the turbidity current generated 7.9 min prior to sampling (on average), and when in $L_6$, the turbidity current was generated 15.4 min earlier. (**b**) The cumulative volume-based particle size distribution $F^v$ shows the sampled suspended sediment during the line *mining pattern* detrained from gravity currents intersected at different times (dashed lines, dark green triangle, and light green diamond), and during two *selfie* experiments (solid lines, red circle, and orange square).

Using carefully planned field experiments, we find it possible to delineate different physical regimes of transport based on in situ data. For example, the suspended sediment distribution measured during the tail end of the *L* maneuver for the *mining pattern* is comparable to that of the *selfie* maneuvers at similar timescales. Investigating further, we present the cumulative volume-based particle size distribution $F^v$ of sediment sampled during the line *mining pattern* and two *selfie* experiments in Fig. 7. During the *mining pattern* line segment, the collector vehicle first intersected, in section $L_1$, detrained sediment from the turbidity current formed along the most recent adjacent track, on average at 7.9 min post-discharge generation. The collector finished the line segment, in section $L_6$, on average at 15.4 min, at that stage sampling older detrained sediment from the turbidity current generated at the start of the previous leg. For comparison, we also look at two *selfie* experiments that were intersected at two quite different times: *selfie* *A*3 encountered the turbidity current at the entry point $\alpha$ at 14.9min, matching the intersection time of section $L_6$. And *selfie* *A*4 left the turbidity current at the exit point $\gamma$ at a much later time at 35 min. The data presented in Fig. 7-b show the cumulative particle size distribution measured at roughly the same time for the *mining pattern*, and the selfies overlap very well, with some small difference in the tail of the distribution due to noise from the low sample in $A3_{\alpha }$. These results suggest that despite the complexity of the *mining pattern* maneuver compared to the *selfie* maneuver, on similar timescales, the sediment left in suspension at 5m above the seabed is remarkably similar. This is a significant result considering that the *mining pattern* maneuver comprises a multitude of adjacent driven collection tracks, with potentially interacting turbidity currents, pickup of freshly deposited sediment layers composed of the recently settled sediment from the turbidity current head, and sediment advected from complex end-of-track maneuvers. We furthermore note that the *mining pattern* experiment was conducted across numerous tidal cycles during stable conditions that were characteristic of the abyssal region in the BGR-E1 area, a different location to the *selfie* experiments, which were carried out in the GSR-B4 area, roughly 400 km away and a few weeks earlier. The implication is that for this region of the CCZ, after several minutes have passed to allow the fastest settling sediment to settle, the particle properties measured above the turbidity current head height become somewhat agnostic to the history of the sediment suspension. Practically, this finding suggests that collecting sediment size and settling velocity data during a simple *selfie* maneuver could be a worthwhile exercise to execute first before starting *mining pattern*-like operations. The PSD data from a *selfie* experiment could then be used to initialize sediment transport models.

The data presented in this paper, sampled at a height of 5 m above the seabed, concern suspended sediment that is understood to have been detrained from a generally low-lying turbidity current plume created by the collector vehicle. This assertion is supported by the low concentration levels of suspended sediment detected by the uppermost turbidity meter, positioned at 3 m above the seabed in Fig. 2-b. Unlike the sediment deposited locally by the turbidity current that forms post-discharge, this detrained sediment will be advected by background currents away from the mining area^5^, potentially causing indirect environmental impact. The PSDs presented herein, using data from the *selfie* and the *L* maneuvers, are likely to be much more appropriate than PSDs obtained from samples of the surface sediment layer. This is crucial because it has been established that the correct choice of PSD is one of the most critical parameters when predicting the extent of sediment plumes in the far field^4^. Initializing a model with a sediment suspension composed predominantly of either more or less fine -slow settling- particles initially would lead to potentially overestimating or underestimating the sediment plume’s lateral extent and overall residence time. At 5 m above the seabed, we found that fractions of fine sediment smaller than 63 $\upmu$m varied significantly between 67 and 84.7$\%$ of the sediment composition by volume across different times. Very different results were previously reported in laboratory-based studies investigating the hydrodynamic properties of CCZ surface seabed sediment^17^. In this case, ex situ particle size measurements were performed on seabed sediment samples obtained with multi-corer in the BGR-E1 area near the location of the *mining pattern* experiment conducted in our trials. To conduct the measurements, the suspended sediment was prepared by continuously stirring oven-dried core sediment extracted from the upper layer of the seabed. This study indicated that fine sediment particles smaller than 63 $\upmu$m occupied between 53 and 73$\%$ of the measured sediment suspension by volume.

Finally, our data also suggest that the settling velocities of the sediment are markedly different from the classical Stokes settling model commonly utilized in sediment analysis, aligning with prior studies on marine particle settling rates^17^. Notably, we find that only two-thirds of particles suspended in the *R* maneuver that are larger than 200 $\upmu$ have settled out by the time the *CO* maneuver is completed, which takes roughly 90 s. Applying Stokes’ law to particles of diameter larger than 200 $\upmu$m, assuming a dry bulk density of 2.6 g/cm$^3$, suggests an anticipated settling distance of 2–3 m within the duration of the *CO* maneuver, implying that all of the sediment within this fraction would be expected to have settled well below the collector height by the time a sample is collected by the LISST-RTSSV. The persistence of a significant fraction of the irregularly shaped medium particles during *CO* is therefore indicative of lower settling velocities than predicted by Stokes’ law and more consistent with the slower settling speeds of aggregates reported in ex-situ experiments, which predict vertical settling of 0.1m over the duration of the *CO* maneuver^17^. This slower settling rate is attributed to shape and other factors, such as particle density, which, in turn, is influenced by composition and morphology. Other factors that could have contributed to the persistence of 200 $\upmu$m particles during *CO* maneuvers include sustained ambient turbulence generated by the collector and the initial suspension heights substantially exceeding that of LISST-RTSSV on the collector; observations from other studies of Patania II have reported some cases of plumes reaching heights up to 8 m^5^. Looking ahead, accurately modeling sediment transport necessitates comprehensive in situ measurements encompassing not just particle size and shape, but ultimately also settling velocity distribution.

## Methods

### LISST-RTSSV sampling procedure and data analysis

**Fig. 8:**
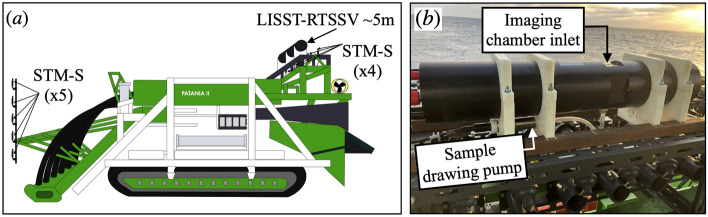
(**a**) Schematic of the pre-prototype nodule collector Patania II, with the mounted LISST-RTSSV at the rear of the vehicle at 5m above the seabed, the locations of the nine Seapoint Turbidity Meters type S (STM-S) are also illustrated, with four STM-S at the rear of the vehicle in the vicinity of the LISST-RTSSV, and five STM-S on a front-mounted pole at heights of 1 m, 1.6 m, 2 m, 2.5 m and 3.2 m above the seabed. (**b**) Photo of the LISST-RTSSV mounted at the rear of the GSR pre-prototype nodule collector vehicle, Patania II.

The LISST-RTSSV was mounted at the rear of the nodule collector vehicle at a height of $\sim$5 m above the seabed. The instrument is secured to the frame of the collector vehicle with electrically insulated bracketing bolted from below (Fig. 8-a). Special care has been given to the positioning of the LISST-RTSSV with respect to neighboring instruments and the frame of the collector vehicle, ensuring that the sampled sediment by the instrument is undisturbed. The LISST-RTSSV instrument was equipped with a Titanium Submersible Pump, which draws a new fluid sample from the top inlet of the imaging chamber. The sampling pump operates for roughly 5 s, during which the typical shear rate experienced by the sediment in the column is less than 0.85 s$^{-1}$. At the typical sediment concentrations encountered in this study, this shear rate is not conducive to flocculation or disaggregation over such a short period of time^17^, and is much less than the O(10)s$^-$ shear rates encountered by the sediment as it passes through and out the rear of the collector. At the time of its deployment, the imaging chamber inlet remained open throughout the sampling process, as the trap door had not been installed yet at this stage of the instrument development. This resulted in an induced flow within the imaging chamber so that the in situ particle settling velocity measurements could not be obtained from this version of the instrument during this deployment. The LISST-RTSSV operated under the burst/sleep mode, pre-configured based on the scheduled collector vehicle operations and the duration of each descent, to satisfy the usage demands of the available local power and onboard internal memory.

### Defining the particles size

Particle size analysis and reporting of PSD is challenging as only objects of simple geometry, spheres to be specific, can be unambiguously described by a unique and well-defined descriptor, namely the diameter. Water-borne particles are, however, irregularly shaped with a distribution of sizes, for which reporting of particle size and size distribution information requires that all pertinent particle information is provided, including particle shape descriptors. The equivalent sphere concept is employed to enable measurements of a “particle size”, where an arbitrary particle size is characterized by the diameter of its equivalent sphere, *D*. Here, non-spherical particles of the same size may yield a size distribution where the smallest size corresponds to the smallest particle cross-section and the largest size to that of the largest cross-section. Under this concept, a collection of the same particles analyzed with different sizing techniques will result in varying equivalent sphere diameters, thus yielding different PSDs for the same particle suspension. The most pertinent equivalent diameter for direct imaging-based sizing techniques is the equivalent projected area diameter^26^, defined as $D^A = \sqrt{4 A/ \pi }$, where *A* is the area of the particle projection in random orientation. $D^A$ is the diameter of a circle having the same area as the particle’s projection. Of note, particles having the same equivalent diameter can have vastly different shapes. It is expected that projected area-based measurements are more precise than linear dimensions and their combinations, and therefore, the particle size defined herein refers to the equivalent projected area diameter $D^A$.

### Splicing SP and LP data and PSD calculation

The LISST-RTSSV in situ camera system, developed herein, is currently one of the most comprehensive direct microphotography systems capable of measuring in situ particle sizes down to 3 $\upmu$m at abyssal depths down to 6000 m. To deal with inherent restrictions of image resolution and restriction of the field of view from up-to-date technology, two different cameras with lenses of different magnifications are employed to cover the required size range, referred to as Small Particle imaging system (SP) and Large Particle imaging system (LP) that result into two particle size distributions which must be spliced following the procedure outlined in this section. Two image examples from the SP and LP imaging system collected during one of the LISST-RTSSV deployments are shown in Fig. S1.

We consider a discrete set of uniformly distributed particle sizes $D^A_i$, such that $D^A_i = i\Delta D$ for $i=0:n_b$. Here, $\Delta D$ is the interval width, and $n_b$ is the total number of intervals. Then, we define $N_i$ as the number of particles per unit volume that have a size in the bin (interval) $[ D^A_i, D^A_i + d D^A ]$. This is calculated as $N_i = n_{i}/v$, where $n_i$ is the number of particles in bin *i* and *v* is the suspension volume (of units $\mu$L), such that the unit of $N_i$ is $\mu$L$^{-1}$. As the imaging system is composed of two cameras, *LP* and *SP*, of different magnifications, two deviations are present in the lower and upper end of the size range. The deviation at the lower end results from the limit of detection set by the resolution of the *LP* camera. At the upper end, the detection limit is set by the small number of particles measured in the large size fractions by the *SP*, making the results for these size fractions statistically unreliable. For simplification, the upper bound is equal to the depth of field of the *SP* camera, $DF^{SP}$, such that the detected particles are considered to be confined within the depth of field. The following procedure is followed to combine the particle number concentration per suspension volume from the small particle camera, $N^{SP}_i$, and the large particle, $N^{LP}_i$:

1 $$\begin{aligned} N_i = \left\{ \begin{array}{ll} N^{SP}_i& D^{SP}_{min} \le D^A_i< D^A_{LP,min} \\[1ex] (N^{SP}_i+ N^{LP}_i)/2 & D^A_{LP,min} \le D^A_i< DF^{SPC} \\[1ex] N^{LP}_i & DF^{SPC} \le D^A_i < DF^{LPC} \end{array} \right. \end{aligned}$$

where $N^{SP}_i = n^{SP}_{i}/v^{SP}$ and $N^{LP}_i = n^{LP}_{i}/v^{LP}$. The suspension volumes $v^{SP}$ and $v^{LP}$ are estimated from $v = FV \times DF$, where *FV* is the camera field of view. This approach yields a good overlap between the results of the two cameras. We introduce the histogram of particle size *p*, where the *i*-th component of *p*, $p_i$, represents the percentage of particles that are in the *i*-th size bin. It is defined component-wise as

2 $$p_{i} = \frac{{N_{i} }}{{\sum\nolimits_{{i = 1}}^{{n_{b} }} {N_{i} } }},\quad i = 1:n_{b} .$$

We additionally introduce the cumulative frequency *F*, i.e., the percentage of particles that are smaller than a certain size. While this can be approximated by the cumulative histogram $P_i = \sum _{j=1}^i p_j$, it is more accurately calculated following the approach outlined in Ref.^34^. This is done by ordering every particle from the smallest to the largest, with the *k*-th particle having a size $D^A_k$, then constructing the cumulative distribution particle by particle (instead of bin by bin), such that the discrete cumulative probability distribution has a *k*-th value $F_k = \frac{k}{n}$, with *n* the total number of particles. For convenience, *F* can then be interpolated to any value $D^A$. So, when considering *F* as a continuous function of particle size $D^A$, then $F(D^A)$ is the percentage of particles that are smaller than $D^A$.

Generally, the quantity of interest in sediment suspensions is the sediment concentration, which does not depend on the number of particles per unit volume, but on the volume of particles per unit volume. So, it is convenient to consider the volumetric histogram of particle size, $p^v$, where $p^v_i$ is the percentage of the total particle volume that is occupied by particles in size bin *i*. It is defined as:

3 $$p_{i}^{v} = \frac{{v_{i} N_{i} }}{{\sum\nolimits_{{i = 1}}^{{n_{b} }} {v_{i} N_{i} } }},\quad i = 1:n_{b} ,$$

where $v_i=\frac{\pi }{6}{D^A_i}^3$ is the mean particle volume in size bin *i*, assuming the particle to be a sphere of diameter $D^A_i$.

As for the cumulative frequency, a volumetric cumulative frequency $F^v$ can be defined. This is again calculated by sorting all particles in increasing order of diameter size $D^A_k$, with $k=1:n$. Then, the *k*-th component of the cumulative frequency $F^v$ is given as $F^v_k = \sum \nolimits _{i=1}^k v_k/\sum \nolimits _{i=1}^n v_k$, where $v_k=\pi {D^A_k}^3/6$. Once again, this can then be interpolated at any particle size $D^A$, and when considering $F^v$ as a continuous function of particle size $D^A$, $F^v(D^A)$ is the percentage of the total particle volume that is occupied by particles smaller than $D^A$.

## Supplementary Information

Supplementary Information.

## Data Availability

The data presented in this study are available on request from the corresponding author.
