# Supplementary material for: In situ optical measurement of particles in sediment plumes generated by a pre-prototype polymetallic nodule collector
Source: Sci Rep. 2024 Oct 12;14:23894. doi: 10.1038/s41598-024-72991-y (PMC11470929; doi:10.1038/s41598-024-72991-y)
Supplement: Supplementary file 1 — Supplementary Information. [file 41598_2024_72991_MOESM1_ESM.pdf]

Supplementary Information (SI)

Characteristics of the LISST-RTSSV and image processing

The LISST-RTSSV *in situ* camera system, developed herein, is currently one of the most comprehensive direct microphotography systems capable of measuring *in situ* particle sizes down to 3μm at abyssal depths. The light source, imaging systems, settling column, and necessary operating electronics are all enclosed in the same housing, designed for use at depths of 6000m. As of today, measurement of the full range of marine particle sizes with diameters ranging from a few microns to several millimeters cannot be satisfied with single camera systems due to inherent restrictions of image resolution and restriction of the field of view. To deal with these limitations, two cameras with lenses of different magnifications are employed to cover the required size range. The first camera, which is dedicated to viewing small particles, is called the Small Particle imaging system (SP). The second camera, the Large Particle Imaging System (LP), is used to measure large particles. The SP imaging system is composed of a monochrome camera with a Sony IMX226 1/1.7" CMOS sensor of resolution 4000$\times$3000 pixels with 1.85 $\times$ 1.85μm pixel size mounted to a 0.5X primary magnification long working distance telecentric lens, giving a field of view of 4.9$\times$3.7mm for a depth of field of DF^{SP} = 2.43mm. The LP imaging system is composed of a monochrome camera with a Sony IMX264 2/3" CMOS sensor of resolution 2448$\times$2048 pixels with 3.45$\times$3.45μm pixel size mounted to a 1.7X primary magnification long working distance telecentric lens. This system yields a field of view of 17.6$\times$14.7mm for a depth equal to DF^{LP}= 5.25mm. Two images captured by the SP and LP imaging system during the LISST-RTSSV deployment are shown in figure S1.

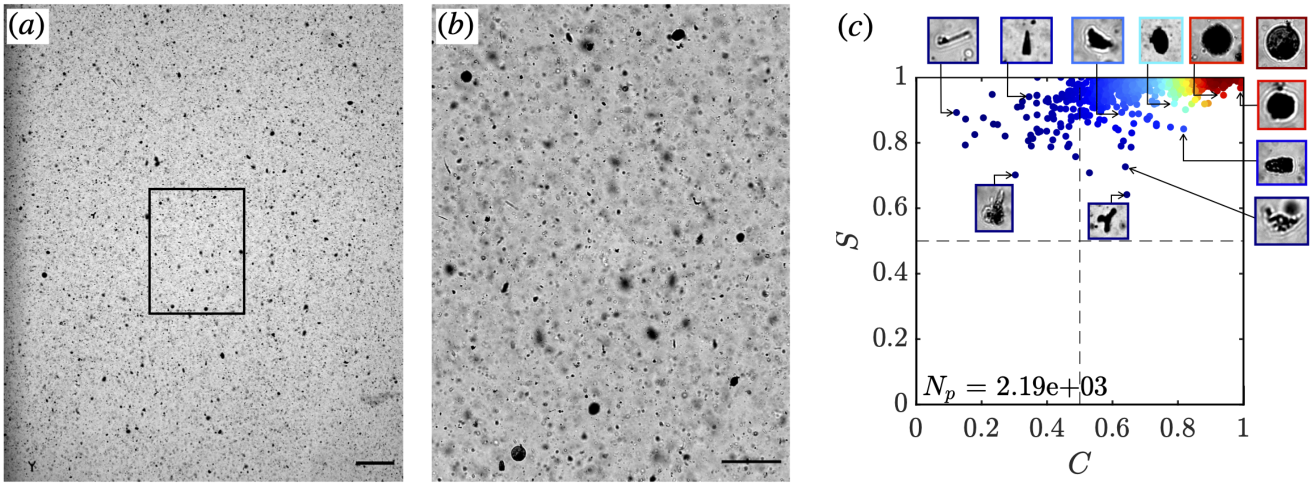

Figure S1. (a) and (b) show images captured by the LP and SP imaging system, respectively, during deployment of the LISST-RTSSV in the course of the operations of the collector vehicle. Scale bars are 1mm and 0.5mm for (a) and (b) respectively. The box in (a) shows the location of the field of view of the SP camera within the LP camera. The shape parameters, solidity S and circularity C extracted from the frames (a) and (b) are presented in Figure (c), where particles of various shapes are given for illustration purposes.

Monochrome cameras have been chosen as they generally offer higher resolution and better light sensitivity, with square pixels, to simplify the implementation of image processing algorithms for sizing the particles. Because the imaging systems must be enclosed in abyssal pressure-rated housing, the lenses associated with the cameras have a long working distance, necessitating a thick, high-strength glass window between the lens and water. Both imaging systems utilize the same highly collimated LED light source. The SP and LP imaging systems are relayed through a set of prisms positioned such that the field of view of the SP camera is contained within that of the LP camera, enabling it to probe the same volume of water. The specifications and the performance of the dual-camera system have been verified using a set of optical bench tests. The SP and LP camera resolution and field of view specifications allow for some overlap in joining the size distributions. To validate the dual-camera imaging system for particle sizing, considerations regarding the illumination, camera, and lens are accompanied by extensive validation using a set of optical bench tests. The resolution of each of the cameras was determined using the Modulation Transfer Function (MTF), which is the measurement of an imaging system's ability to transfer contrast at a particular resolution from the object to the image, such that resolution and contrast can be combined as a single specification[35–37]. Based on vanishing resolution contrast criteria of 20%, the smallest resolvable size is approximately 10μm for the LP system and 1μm for the SP system. More confidently, 15μm and 3μm objects and bigger are resolved with contrast values higher than 60%, respectively, for the LP and SP.

The depth of field (DoF) of each imaging system was obtained with a meticulous calibration procedure consisting of using line targets with lines of known frequency resolution tilted at a known angle. The DoF is then deduced from knowing the distance where the line grating appears to be in focus, followed by a geometrical transformation accounting for the target's tilt with respect to the camera. Of note, the MTF and the DoF are unique to a specific configuration for each component in the imaging system (camera sensor, lens, and illumination). Any changes introduced to the camera settings or distances between the optical components will yield different numerical values. The DoF is required to correctly calculate the imaging control volume necessary to splice the size distributions obtained from the two imaging systems and to calculate the volumetric and mass concentrations. Calculations of concentrations were validated through various suspensions of NIST traceable particle standards of known concentrations.

During the sampling, the images from the SP and LP imaging systems are saved in TIFF file format with LZW loss-less compression for no image quality degradation and stored locally on an internal hard drive. The cameras were limited to 8 bits to reduce the size of the images captured without deteriorating image quality. Upon retrieving the collector vehicle, the images collected by the LISST-RTSSV are downloaded and sorted according to relevant collector vehicle operations before image analysis. The principles for image analysis follow three main steps: restoration, segmentation, and measurement. Restoration involves improving the image quality by equalizing the background light intensity and filtering out image noise across all images. Segmentation consists of separating in-focus sharp particles from the background and out-of-focus particles using a threshold level. The resultant binary image (white particle regions on a black background) from the thresholding segmentation contains particle information ready for image measurement, where particle parameters of interest are extracted for sizing and counting. The number of pixels contained in each individual white region in the resultant binary image is equivalent to the particle projected area A taken in a random orientation. Several frames recorded throughout this deployment contained images of microscopic living organisms, which were eliminated from the particle data presented in post-processing.

Calibration of Seapoint Turbidity Meters

The suspended sediment concentration data was obtained from Seapoint Turbidity Meters type S (STM-S), referenced STMS-18100 to STMS-18108, mounted at the front and the rear of the collector vehicle. These STM-S underwent two calibrations in total. The first calibration is a factory calibration that aims to document potential instrument drift and any temporal variability in the sensor's response to changes in concentration and compare the sensors' sensitivity, linearity, and reproducibility. The second calibration for suspended sediment concentration (SSC), which was conducted in the laboratory before the field studies, establishes a relationship between the suspended sediment concentration and the raw turbidity signal output (in counts); see detailed calibration protocol for the SSC in[5]. The linear calibration curve for every STM sensor used in the field studies is presented in Figure S2. The slope was obtained by minimizing $C-(a\times S)$, where C is the known concentration of the calibration sample in mg/l, a is the slope of the linear calibration curve to be determined, and S is the raw output signal from the turbidity sensor in counts. The zero output for a blank sample was verified for the turbidity sensors using black tape over the windows. It corresponded to within ~ 0.1 counts of the ambient turbidity output of the STM sensors in the undisturbed water of the abyssal ocean.

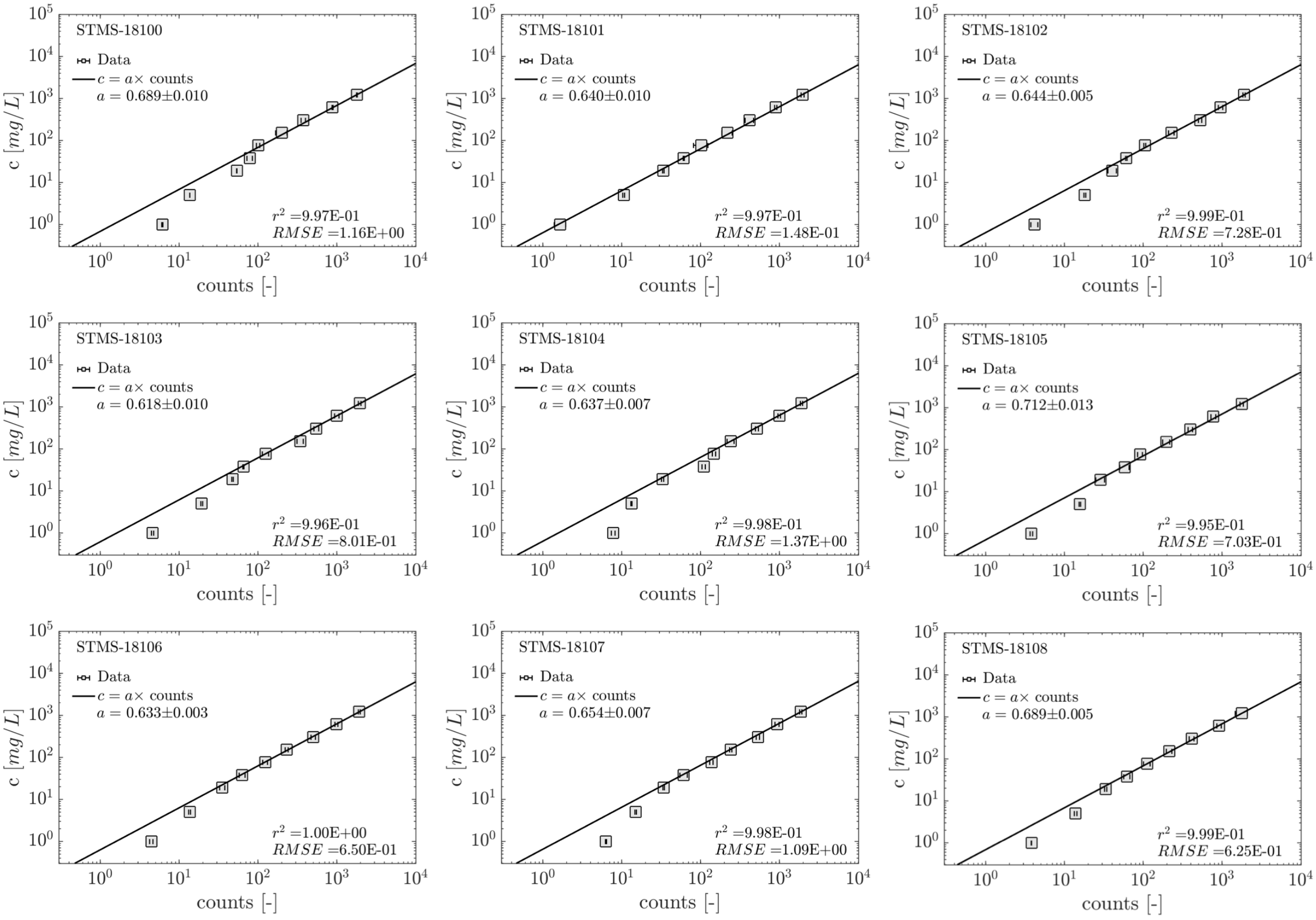

Figure S2. STM-S calibration data and corresponding slope, a, to the linear fit for each turbidity sensor used during the field studies on the collector vehicle. The coefficient of determination r^2 and the root mean squared error $RMSE$ the linear fits are reported.

Location of the collected samples during the selfie maneuvers

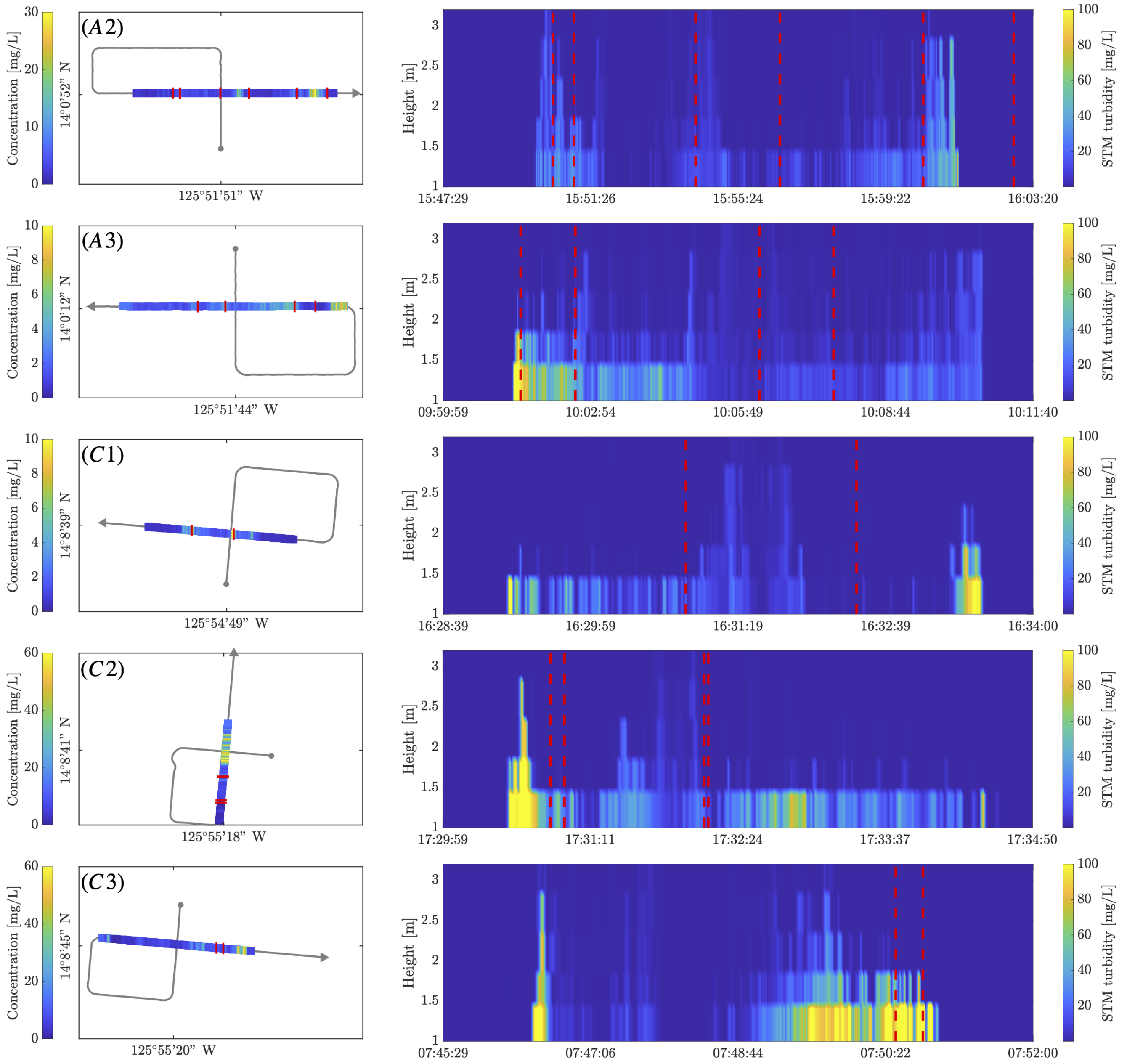

Figure S3. (Left) Top view of the trajectory of all conducted selfies with LISST-RTSSV sampling locations, with the colormap corresponding to the averaged turbidity in mg/L measured with the Seapoint turbidity meter sensors positioned at the back of the collector vehicle at $5m$ above seabed. (Right) Colorplot of the vertical profiles of concentration as a function of time. The vertical dashed lines indicate the LISST-RTSSV sampling locations. The sediment concentration in mg/L is measured by calibrated Seapoint turbidity meters positioned in the front of the collector vehicle.

Data collected during the selfie maneuvers

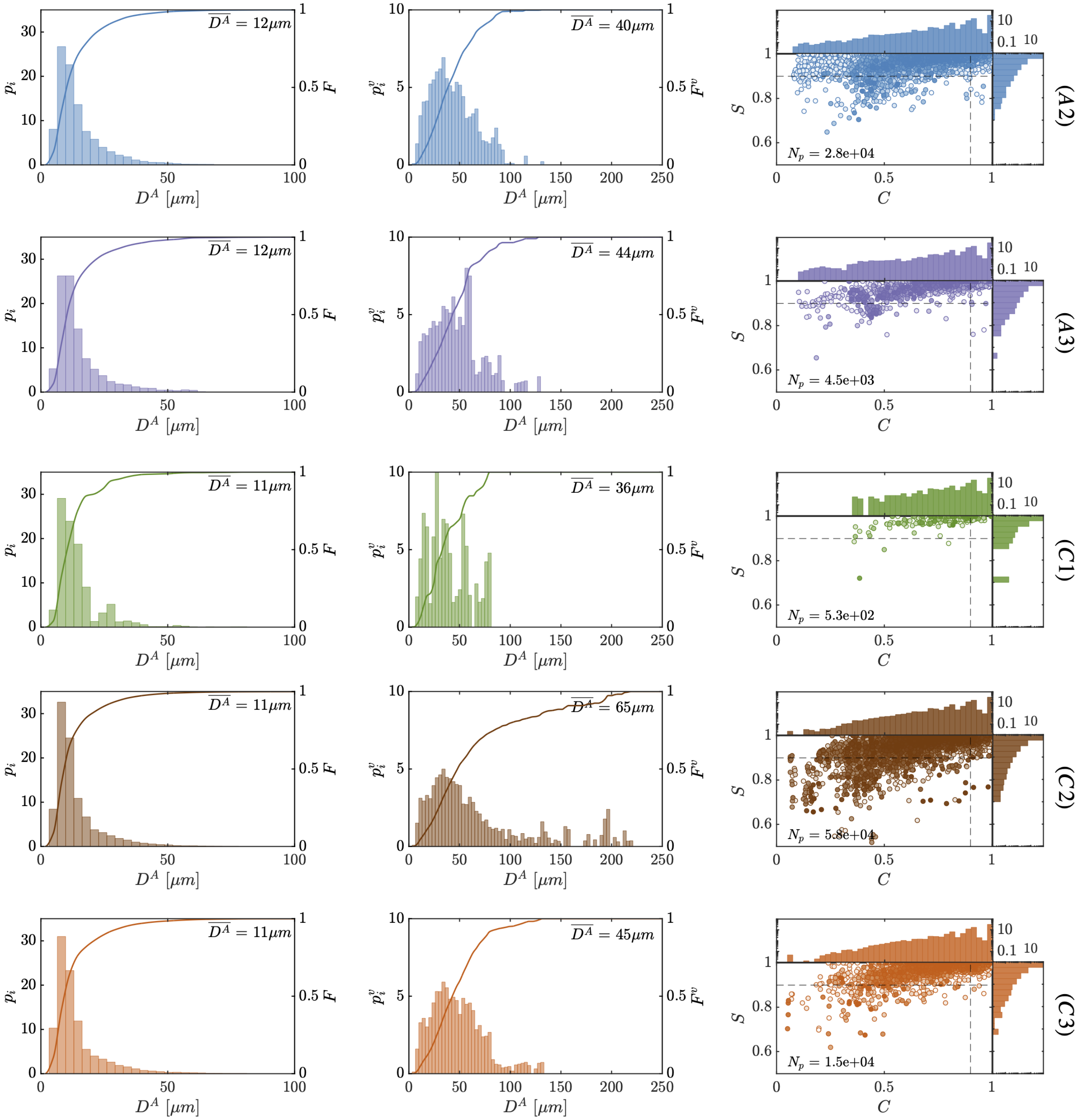

Figure S4. Shows the number based particle size distributions, p_i, and the relevant particle shape descriptors, solidity S versus circularity C. (a) and (b) correspond to the selfies $(A2)$ and $(A3)$ conducted in area A, and (c), (d), and (e) to selfies $(C1)$, $(C2)$ and $(C3)$, respectively.

Data collected during the mining pattern experiment classified by maneuver type

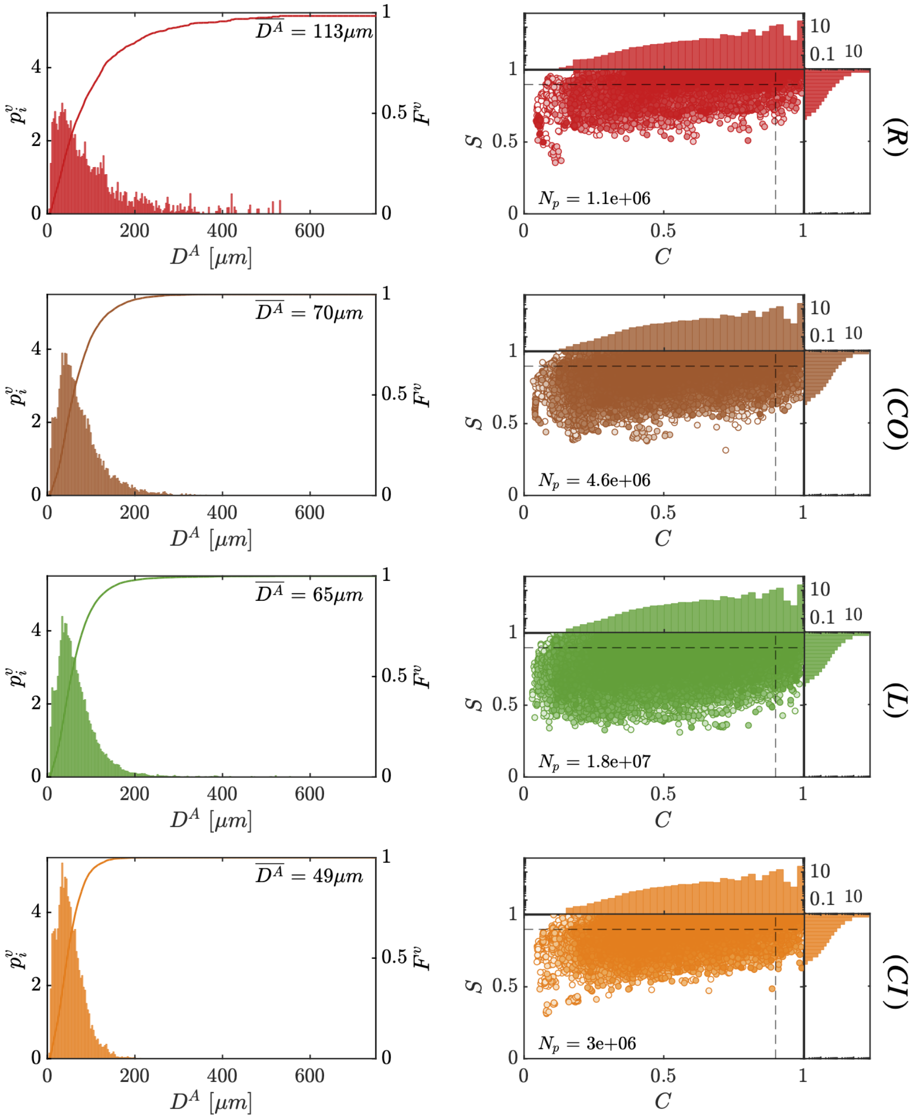

Figure S5. Shows the volume-based particle size distributions p_i^v with the cumulative volume-based particle size distribution F^v, and the relevant particle shape descriptors, solidity S versus circularity C, of the sampled suspended sediment generated by the different operations during the mining pattern maneuvers (R, CO, L and CI).

Data collected during the line driving maneuver categorized by line section

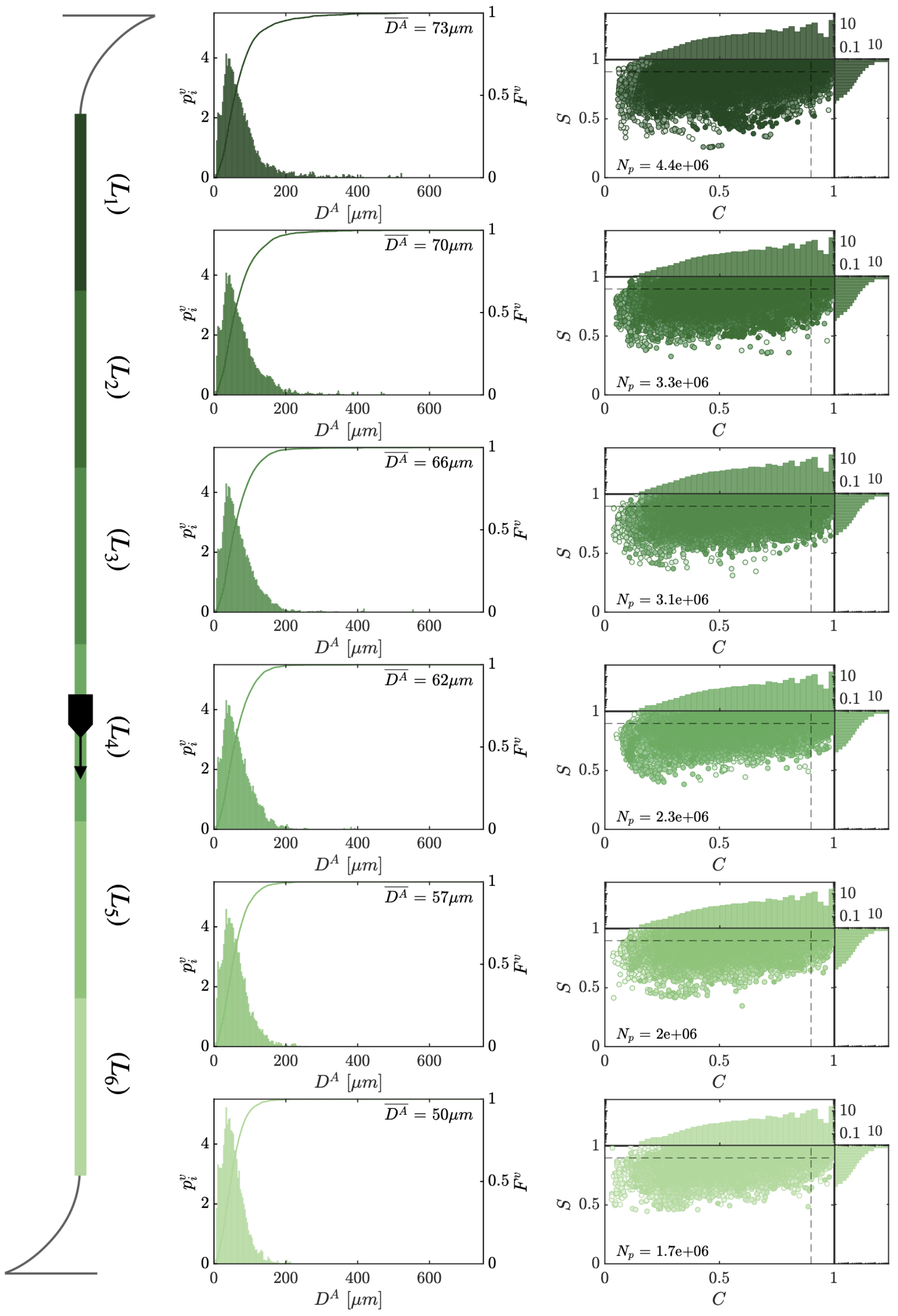

Figure S6. Shows the volume-based particle size distributions p_i^v with the cumulative volume-based particle size distribution F^v, and the relevant particle shape descriptors, solidity S versus circularity C, of the sampled suspended sediment sampled along the mining pattern line.
